# The growth inhibitory effect of human gingiva-derived mesenchymal stromal cells expressing interferon-β on tongue squamous cell carcinoma cells and xenograft model

**DOI:** 10.1186/s13287-019-1320-z

**Published:** 2019-07-29

**Authors:** Lingqian Du, Qianyu Liang, Shaohua Ge, Chengzhe Yang, Pishan Yang

**Affiliations:** 1 Department of Stomatology, The Second Hospital of Shandong University, Jinan, 250033 Shandong People’s Republic of China; 2 Shandong Provincial Key Laboratory of Oral Tissue Regeneration, School of Stomatology, Shandong University, 44 West Wenhua Road, Jinan, 250012 Shandong People’s Republic of China; 3 Department of Periodontology, School of Stomatology, Shandong University, Jinan, People’s Republic of China; 4 Department of Oral & Maxillofacial Surgery, Qilu Hospital and Institute of Stomatology, Shandong University, 107 Wenhua Road West, Jinan, 250012 Shandong People’s Republic of China

**Keywords:** Gingiva-derived mesenchymal stromal cell, Interferon-β, Tongue squamous cell carcinoma, Proliferation

## Abstract

**Background:**

Interferon-β (IFN-β) is a cytokine with pleiotropic cellular functions, including antiviral, antiproliferative, and immunomodulatory activities. IFN-β inhibits multiple tumor cell growth in vitro. However, the contradiction between the therapeutic dose of IFN-β and its maximally tolerated dose is still inextricable in vivo. Human gingiva-derived mesenchymal stromal cells (GMSCs) represent promising vehicles for cancer gene therapy. This study evaluated the potential of GMSCs genetically engineered to produce IFN-β as a targeted gene delivery system to treat tongue squamous cell carcinoma (TSCC) in vitro and in vivo.

**Methods:**

A lentiviral vector encoding IFN-β was constructed and transfected into GMSCs to obtain IFN-β gene-modified GMSCs (GMSCs/IFN-β). Enzyme-linked immunosorbent assay (ELISA) was used to measure the IFN-β concentration in conditioned medium (CM) from GMSCs/IFN-β. The Cell Counting Kit-8 (CCK8), colony formation assay, and flow cytometry were used to detect the effects of GMSCs/IFN-β on TSCC cell line CAL27 cell growth and apoptosis in vitro. TSCC xenograft model was developed by subcutaneous injection of CAL27 cells into BALB/c nude mouse, and the role of intravenously injected GMSCs/IFN-β in engrafting in TSCC and controlling tumor progression was measured in vivo.

**Results:**

GMSCs/IFN-β expressed a high level of IFN-β. Both CCK8 and colony forming assay showed that GMSCs/IFN-β significantly inhibited the proliferation of CAL27 cells compared with the GMSCs, GMSCs/vector, or DMEM group. Flow cytometry analysis demonstrated that the CAL27 cell apoptosis rate was higher in the GMSCs/IFN-β group than in the other three groups. The in vivo experiment revealed that GMSCs/IFN-β engrafted selectively in TSCC xenograft and expressed a high level of IFN-β. There were smaller tumor volume and lower number of Ki67-positive cells in the GMSCs/IFN-β group than in the GMSCs, GMSCs/vector, or phosphate-buffered saline (PBS) group. Interestingly, GMSCs and GMSCs/vector also presented the potential of CAL27 cell growth inhibition in vitro and in vivo, although such an effect was weaker than GMSCs/IFN-β.

**Conclusions:**

GMSCs/IFN-β inhibits the proliferation of TSCC cells in vitro and in vivo. These results provide evidence that delivery of IFN-β by GMSCs may be a promising approach to develop an effective treatment option for TSCC therapy.

## Background

Oral squamous cell carcinoma (OSCC) is a common cancer, and it is estimated that there were almost 354 million new OSCC cases and 177 million associated deaths worldwide in 2018 [1]. Tongue squamous cell carcinoma (TSCC) is the most common type of OSCC with worse prognosis, especially for the advanced cases. Even without any clinical signs of metastasis, up to 30% of all TSCC patients have histologically detectable spread to the lymph nodes. Currently, therapy for locally advanced TSCC is complex, including surgical resection and postoperative radiation [2]. Among the therapies, biotherapy is the vitally important one which hopefully improves the survival rate. But a safe and effective biotherapy strategy is still unavailable to date.

Interferon-β (IFN-β), a therapeutically attractive member of the IFN family, is one of the classic tumor suppressors, which has been proven to significantly inhibit tumor cell growth and induce apoptosis in vitro [3, 4]. The new mechanisms by which IFN-β exerts its anticancer effect have also been explored [5, 6]. However, systematic administration of recombinant IFN-β could not generate and maintain therapeutic dose in the tumor sites due to its short half-life [7]. The requisite concentrations of IFN-β to inhibit the growth of tumor cells in vivo are higher than the patients maximally tolerated dose [8]. Therefore, systematic administration of recombinant IFN-β fails to inhibit tumor cell growth in vivo and has shown poor effect in clinical trials. IFN-β gene therapy employing adenoviral vectors is effective in lung cancer on account of the continuous secretion of IFN-β by infected cells [9]. However, viral vector-based gene delivery administration has been limited by vector safety, organ toxicity, and difficulty in selective delivery of the therapeutic gene [10]. Therefore, the contradiction between the therapeutic dose and maximally tolerated dose is still inextricable.

Mesenchymal stromal cells (MSCs) can be recruited to the tumor site and are promising vehicles for therapeutic cytokine gene delivery [11–13]. IFN-β gene-modified MSCs via systemic administration have also been demonstrated to successfully migrate to the tumor environment and attenuate tumor growth in experimental animal models such as hepatocellular carcinoma, bronchioloalveolar carcinoma, and melanoma [14–16]. However, the promotive or inhibitory effects of MSC engrafting into the tumor environment on TSCC growth have not well been demonstrated. Although bone marrow (BM) has been the most commonly used source of MSCs, one study demonstrated that human BMSCs can induce collagen production and tongue cancer invasion [17]. Contrarily, human gingiva-derived mesenchymal stromal cells (GMSCs) are reported to suppress oral cancer cell growth in vitro and in vivo [18]*.* Moreover, GMSCs display stable phenotype and telomerase activity in long-term culture, are not tumorigenic, and are easily obtained from the oral cavity with minimal discomfort [19, 20]. However, the strategy that uses GMSCs for delivering a therapeutic gene to TSCC has seldom been investigated [12].

In this study, a lentiviral vector encoding IFN-β was constructed and transfected into GMSCs to investigate the inhibitory effects of GMSCs/IFN-β on TSCC cells in vitro and explore the role of GMSCs/IFN-β in controlling tumor progression in TSCC xenograft model in vivo.

## Materials and methods

### Cell lines

Human TSCC cell line CAL27 cell was obtained from the Shandong Provincial Key Laboratory of Oral Tissue Regeneration (Shandong, China) and cultured in basic medium [Dulbecco’s modified Eagle’s medium (DMEM; Hyclone, SH30243.01) supplemented with 10% fetal bovine serum (FBS; Biological industries, 04-001-ACS) and 50 μg/mL streptomycin with 50 U/mL penicillin G (Sigma-Aldrich, MO, USA)] in a humidified incubator at 37 °C with 5% CO_2_.

### Isolation and identification of human GMSCs

Human gingival tissues were obtained from patients undergoing crown lengthening surgery with no history of periodontal disease at the Department of Stomatology, the Second Hospital of Shandong University. The study protocol was approved by the Medical Ethical Committee of the Second Hospital of Shandong University [Protocol Number: KYLL-2017(LW) 019], and written informed consent was obtained from every patient. Human GMSCs were isolated and characterized using the methods described in our previous study [21]. Briefly, the gingival tissues were minced and digested in 3 mg/mL collagenase type I (Beijing Solarbio Science & Technology, C8140) and 4 mg/mL Dispase II (Roche Diagnostics, 04942078001) for 2 h at 37 °C. After that, the dissociated cell suspension was filtered through a 70-μm cell strainer, transferred to 6-well plates, and cultured in basic medium. Finally, the limiting dilution method was used to purify GMSCs from the primary cells. GMSCs at passage 3 were subjected to flow cytometry analysis and evaluations of osteogenic and adipogenic differentiation. GMSCs were incubated with FITC-conjugated mouse monoclonal antibodies specific for human CD73, CD166 (Becton Dickinson Biosciences, CA, USA), and CD90 (R&D Systems, Inc., MN, USA); CD44, CD105, CD14, CD34, and CD45 (eBioscience, CA, USA); or isotype-matched control immunoglobulin Gs. Flow cytometry was performed using an Epics-XL/MCL flow cytometer (Beckman Coulter, CA, USA). At least 1 × 10^4^ events were recorded. For osteogenic differentiation, GMSCs were cultured in an osteogenic inductive medium [basic medium containing 50 mg/L vitamin C, 0.1 μM dexamethazone, and 10 mM β-sodium glycerophosphate (Solarbio)]. After 28 days of incubation, mineral deposition was detected by Alizarin Red (Sigma-Aldrich, A5533-25G) staining. For adipogenic differentiation, GMSCs were cultured in an adipogenic inductive medium [basic medium containing 0.5 mM 3-isobutyl-ethylxanthine, 0.5 μM hydrocortisone, 2 mM insulin, and 60 μM indomethacin (Solarbio)]. And after 28 days of incubation, the presence of lipid drops was detected by staining the cells with Oil Red O (Solarbio, G1260). The cultured GMSCs at passages 3–5 were used for the subsequent experiments.

### Construction of IFN-β gene-modified human GMSCs

A lentiviral vector encoding IFN-β (Ubi-MCS-3FLAG-SV40-EGFP-IRES-puromycin-IFN-β) was constructed (Shanghai GeneChem Co., Ltd., Shanghai, China) and transfected into human GMSCs to obtain GMSCs/IFN-β. Retroviral production and infection were performed according to the standard protocol. Briefly, GMSCs were plated at a density of 2 × 10^5^ cells per well in 6-well plates. Parallel control cultures of GMSCs were transfected with blank vector (Ubi-MCS-3FLAG-SV40-EGFP-IRES-puromycin). After 24 h, the cultures were washed with 10 mM PBS (Solarbio, P1020). Then, the lentiviral particles together with 5 μg/mL polybrene (GeneChem) was added to infect the cells and incubated for 8 h. After that, the cells were cultivated in a selection medium containing 0.6 μg/mL puromycin (GeneChem) for 3 days and expanded to obtain a similar population of cells used for the following experiments. To determine the transduction efficiency of IFN-β in GMSCs, cells and conditioned media (CM) were collected and detected by quantitative real-time polymerase chain reaction (qRT-PCR; TaKaRa Bio Inc., RR047A) and enzyme-linked immunosorbent assay kits (ELISA; Thermo Scientific, 414101) for IFN-β in mRNA and protein level at 1 week after infection.

### Collection of GMSC-, GMSC/vector-, and GMSC/IFN-β-conditioned media

TSCC cell line CAL27 cells were cultured with CM derived from GMSCs, GMSCs/vector, or GMSCs/IFN-β to detect the biological effects on the cells. The normal growth media were conditioned by culturing GMSCs, GMSCs/vector, or GMSCs/IFN-β for 24 h [14]. Briefly, GMSCs, GMSCs/vector, or GMSCs/IFN-β at the same passage were seeded at 2 × 10^5^ cells per well in 6-well plates and cultured in a basic medium for 2 days as described above in a humidified atmosphere. After that, the cells were rinsed three times with PBS and cultured with a fresh basic medium for 24 h. The CM was harvested and stored at − 80 °C until use. The basic medium served as the control group.

### Cell proliferation assay

CAL27 cells were seeded in 96-well plates at a density of 1 × 10^3^ cells per well and cultured in basic media. After 24 h, CM or basic media were applied to the CAL27 cells and changed every day. CAL27 cell proliferation was assayed by the Cell Counting Kit-8 (CCK8) according to the manufacturer’s instructions (Dojindo Laboratories, CK04). From the second day on, at the scheduled time points, the tested wells were supplemented with 100 μL DMEM containing 10 μL CCK8, and the plates were incubated for 2 h at 37 °C. The absorbance was measured at a wavelength of 450 nm. All experiments were performed in triplicate and repeated at least two times.

### Colony formation assay

CAL27 cells were seeded in 6-well plates at a density of 1 × 10^3^ cells per well and cultured in basic media. After 24 h, the CM or basic media were applied to the CAL27 cells and changed every day. After 7 days of incubation, the cultures were washed twice with PBS and the colonies were stained with 1% crystal violet (Solarbio) after fixation and counted under microscopic fields (× 40). Aggregates of 50 or more cells were scored as a colony. All experiments were performed in triplicate and repeated at least two times.

### Flow cytometry for cell apoptosis analysis

CAL27 cells were seeded in 6-well plates at a density of 2 × 10^5^ cells per well and cultured in basic media. After 24 h, the CM or basic media were applied to the CAL27 cells and changed every day. After 3 days of incubation, the cells were labeled with Annexin V-FITC/PI staining according to the manufacturer’s instruction (Beyotime Biotechnology, C1052). Then, the cells were harvested and processed for cell apoptosis analysis using flow cytometry. At least 1 × 10^4^ events were recorded. The rate of early apoptosis (Annexin V-FITC^+^/PI^−^) and late apoptosis (Annexin V-FITC^+^/PI^+^) was analyzed. All experiments were performed in triplicate and repeated at least two times.

### Xenografted tumor model

A total of 34 male BALB/c nude mice (5 weeks of age, 15–19 g) were purchased from Beijing Vital River Laboratory Animal Technology Co., Ltd. (Beijing, China). All mice were maintained in the animal care facility at the Second Hospital of Shandong University, and all experimental procedures involving animals were conducted according to the institutional ethical guidelines for animal experiments. This study was approved by the Medical Ethical Committee of the Second Hospital of Shandong University [Protocol Number: KYLL-2017(LW) 020]. The 2 × 10^6^ CAL27 cells were subcutaneously inoculated on the right flank nearly axillary of each nude mouse. Before inoculation, CAL27 cells were trypsinized and suspended in PBS at a concentration of 1 × 10^7^/mL. Two nude mice were used for identification of the tumor development by hematoxylin and eosin (H&E; Solarbio) staining. The 32 nude mice were divided into four groups: PBS treatment group (*n* = 8), GMSCs treatment group (*n* = 8), GMSCs/vector treatment group (*n* = 8), and GMSCs/IFN-β treatment group (*n* = 8). When tumor volume reached 50 mm^3^, 200 μL PBS or 1 × 10^6^ cells in 200 μL PBS were injected into the tail vein. All the injections were conducted according to the specification groups described above twice with 2 weeks interval [22]. After the first inoculation, tumor growth was examined every 3 days by measuring the length and width using a caliper, and the volume of each tumor was calculated as 0.5 × length × width^2^ [16]. On day 28, all the nude mice were sacrificed, and tumors were excised and weighed.

### Immunohistochemistry

The tumor tissues were immersion fixed in 4% paraformaldehyde for 24 h and then embedded in paraffin, sectioned into 4 μm sections, and stained with H&E by standard methods. The number of Ki67-positive cells was detected to examine the cell division in tumor tissues. The sections were incubated with 3% H_2_O_2_ in PBS for 30 min to inactivate endogenous peroxidases after deparaffinization and rehydration and then incubated in PBS containing 1% bovine serum albumin for 30 min at room temperature to block nonspecific staining. After that, the sections were incubated with polyclonal anti-Ki67 antibody (1:200 dilution; Abcam, ab16667) for 1 h, followed by goat anti-rabbit secondary antibody (1:100 dilution; Abcam, ab6721) for 1 h at room temperature. Immunoreactions were detected with diaminobenzidine (DAB; Solarbio, DA1010). The number of Ki67-positive cells in six randomly selected sections was calculated by counting four nonoverlapping microscopic fields randomly.

### Determination of GMSC homing to xenografted tumors

GMSC homing to tumor tissues in vivo was determined by visualization of GMSCs/IFN-β and GMSCs/vector based on fluorescence of enhanced green fluorescent protein (EGFP). Fresh tumor tissues were placed in freezing microtome (Leica CM1950, Germany) and embedded by optimal cutting temperature compound (Sakura, USA). Frozen tumor tissues were then sectioned into 4 μm slices, and the cell nucleus was stained by 4′,6-diamino-2-phenylindole (DAPI). The slices were observed under confocal laser scanning microscopy (ZEISS, LSM 800, Germany) to monitor the EGFP expression. In order to further confirm whether GMSCs can migrate to TSCC xenografts, paraffin sections were made to detect the expression of IFN-β in tumor tissues by immunohistochemical staining. The sections were incubated with polyclonal anti-IFN-β (1:200 dilution; Origen, TA306437) for 1 h, followed by goat anti-rabbit secondary antibody (1:100 dilution; Abcam, ab6721) for 1 h at room temperature. Immunoreactions were detected with DAB. The number of IFN-β-positive cells in six randomly selected sections was calculated by counting four nonoverlapping microscopic fields randomly.

### Statistical analysis

Data were presented as mean ± standard deviation (SD). SPSS software (version 13.0; Armonk, NY, USA) was used for this statistical analysis. Comparisons between the groups for statistical significance were performed with one-way analysis of variance (ANOVA) with a least significant difference test. *P* < 0.05 was considered as significant difference.

## Results

### Isolation and characterization of human GMSCs

**Fig. 1:**
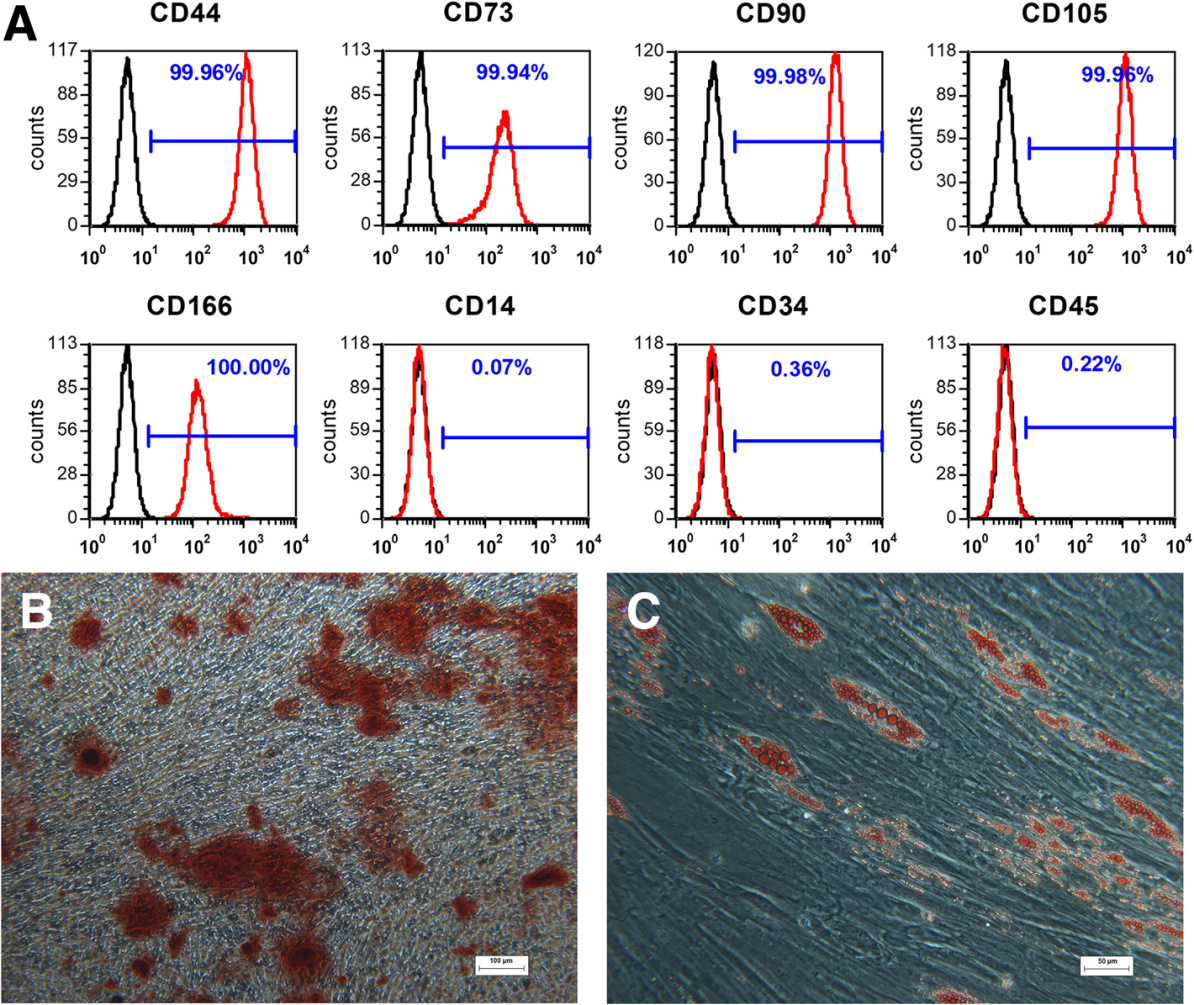
Isolation and identification of human GMSCs. **a** Cell surface markers (CD44, CD73, CD90, CD105, CD166, CD14, CD34, and CD45) were identified through flow cytometric analysis. **b** Representative image of mineralized cell aggregates stained with Alizarin Red following 28 days of osteogenic induction. **c** Representative image of lipid droplets stained with Oil Red O following 28 days of adipogenic induction

We successfully isolated human GMSCs from normal gingival tissues. To identify the characteristics of GMSCs, the surface markers for GMSCs were quantified by flow cytometry. The results showed that GMSCs were positive for MSC-associated makers CD44, CD73, CD90, CD105, and CD166 and were negative for the hematopoietic stem cell markers CD14, CD34, and CD45 (Fig. 1a). To evaluate the multilineage differentiation potential of GMSCs, osteogenic and adipogenic capacities were evaluated. After 28 days of culture in osteogenic inductive medium, GMSCs showed formation of mineralized aggregates and calcium mineralization which was confirmed by Alizarin Red staining (Fig. 1b). After 28 days of culture in adipogenic inductive medium, GMSCs showed formation of lipid droplets that were positively stained with Oil Red O (Fig. 1c).

### GMSCs/IFN-β expressed high level of IFN-β without cellular morphology change

**Fig. 2:**
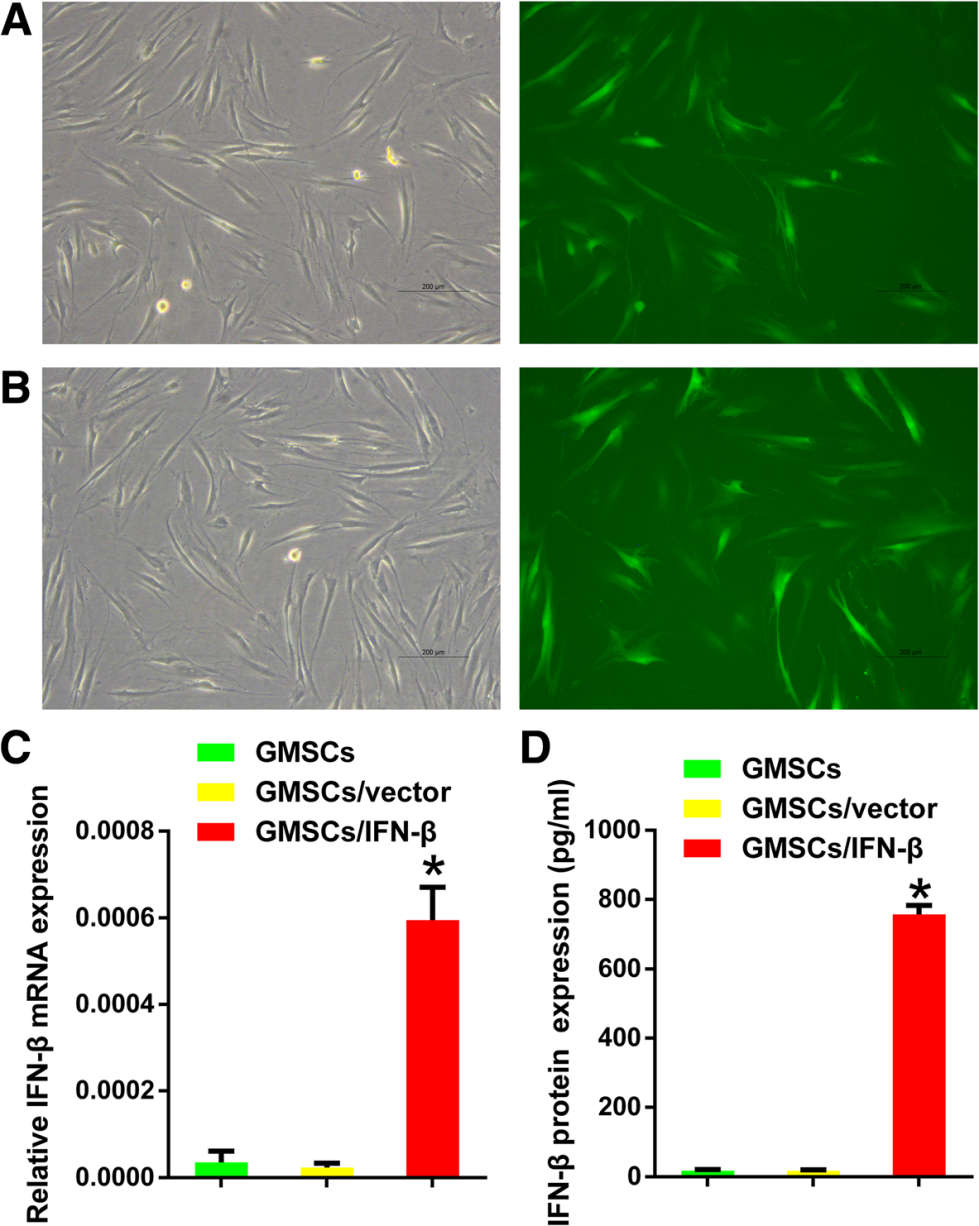
IFN-β was stably expressed in human GMSCs after transfection with IFN-β lentiviral vectors. **a** GMSCs/vector exhibited typical fibroblast-like morphology under a microscope (left). GMSCs/vector had strong green fluorescence expression 48 h after transfection under a fluorescence microscope (right). **b** GMSCs/IFN-β showed no morphology (left) or green fluorescence expression (right) change compared with GMSCs/vector. **c** Relative mRNA levels of IFN-β were significantly increased in GMSCs/IFN-β compared with GMSCs or GMSCs/vector. **d** The concentration of IFN-β protein was significantly increased in the media of the GMSCs/IFN-β group compared with the GMSCs or GMSCs/vector group. All data are shown as mean ± SD. *P < 0.01 versus the GMSCs/vector group

Human GMSC exhibited a fibroblast-like morphology. There was no change in cell shape for IFN-β gene-modified GMSC. Fluorescence microscope observation displayed that both GMSCs/IFN-β and GMSCs/vector had strong green fluorescence expression 48 h after transfection (Fig. 2a, b). Cells and CM were collected to determine the transfection efficiency of IFN-β in GMSCs. IFN-β gene expression and protein secretion were detected by qRT-PCR and ELISA, respectively. The result showed that IFN-β mRNA expression level in the GMSCs/IFN-β group was significantly higher than that in the GMSCs or GMSCs/vector group (*P* < 0.01) (Fig. 2c). IFN-β concentration in CM was 756.7 ± 19.914 pg/mL for the GMSCs/IFN-β group, which was significantly higher than that in the GMSCs or GMSCs/vector group (*P* < 0.01) (Fig. 2d). The expression level of IFN-β displayed no significant difference between the GMSCs and GMSCs/vector groups.

### GMSCs/IFN-β significantly attenuated TSCC cell proliferation in vitro

**Fig. 3:**
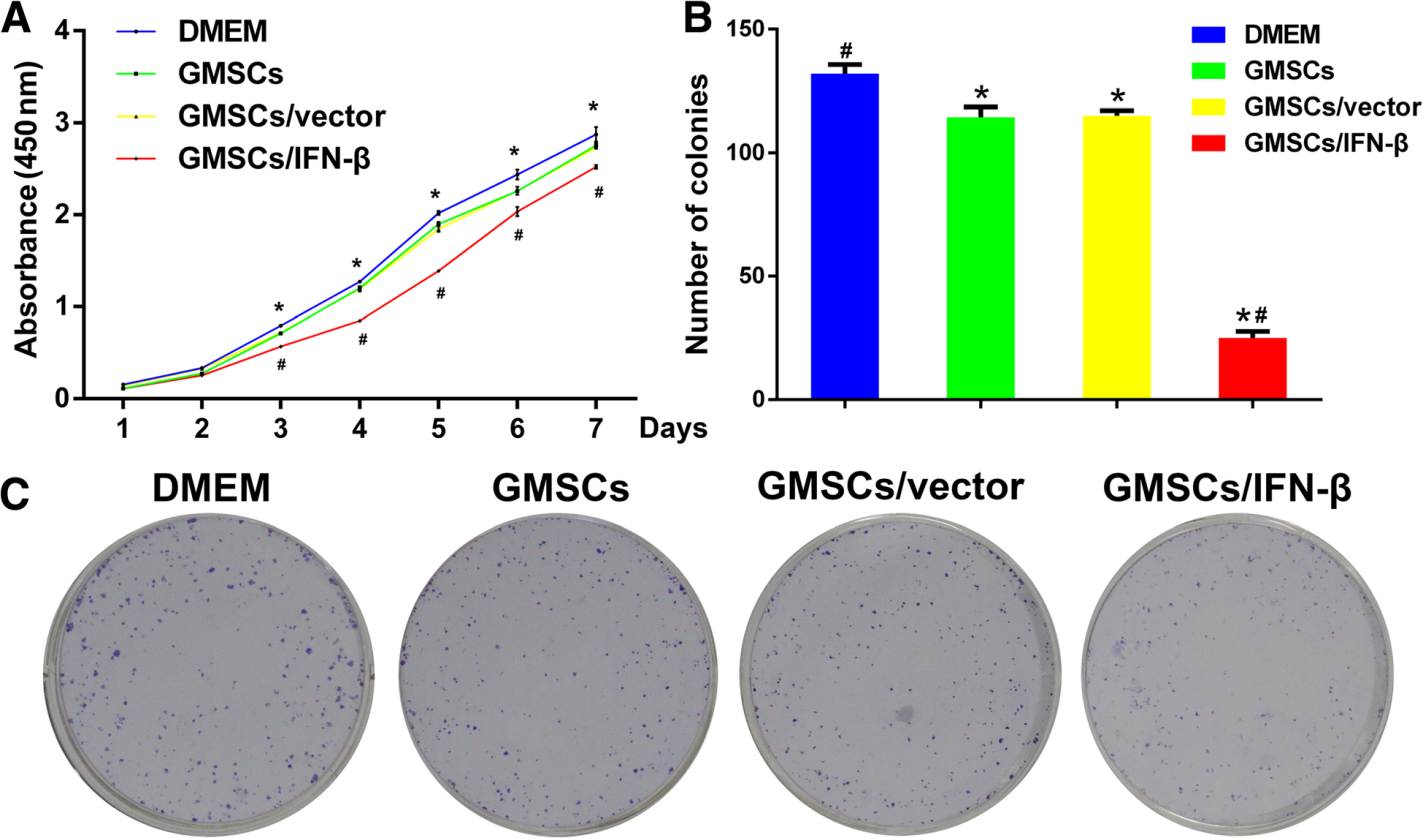
GMSCs/IFN-β inhibited the proliferation of TSCC cell line CAL27 cells in vitro. **a** GMSCs/IFN-β significantly reduced the growth of CAL27 cells compared with the DMEM, GMSCs, or GMSCs/vector group as determined by CCK8 assay. **b** Representative quantification of colonies formed by CAL27 cells in different groups. GMSCs/IFN-β significantly reduced the number of colonies formed by CAL27 cells compared with the DMEM, GMSCs or, GMSCs/vector group as detected by colony formation assay. **c** Representative images of colonies formed by CAL27 cells. All data are shown as mean ± SD. *P < 0.01 versus the DMEM group, ^#^*P* < 0.01 versus the GMSCs/vector group

CCK8 assay showed a significant reduction of CAL27 cell proliferation in the GMSCs, GMSCs/vector, and GMSCs/IFN-β groups compared with the control group after the second day (*P* < 0.01) (Fig. 3a). No significant difference between the GMSCs and GMSCs/vector groups was found. GMSCs/IFN-β dramatically reduced CAL27 cell proliferation compared with the GMSCs or GMSCs/vector group (*P* < 0.01). This proliferation inhibitory activity of GMSCs/IFN-β was further demonstrated with the colony formation assay. The number of colonies detected in the GMSCs, GMSCs/vector, or GMSCs/IFN-β group was decreased compared with the control group (*P* < 0.01) (Fig. 3b, c). Moreover, fewer colonies were seen in the GMSCs/IFN-β group than in the GMSCs or GMSCs/vector group (*P* < 0.01). The number of colonies displayed no significant difference between the GMSCs and GMSCs/vector groups.

### GMSCs/IFN-β induced apoptosis of TSCC cells

**Fig. 4:**
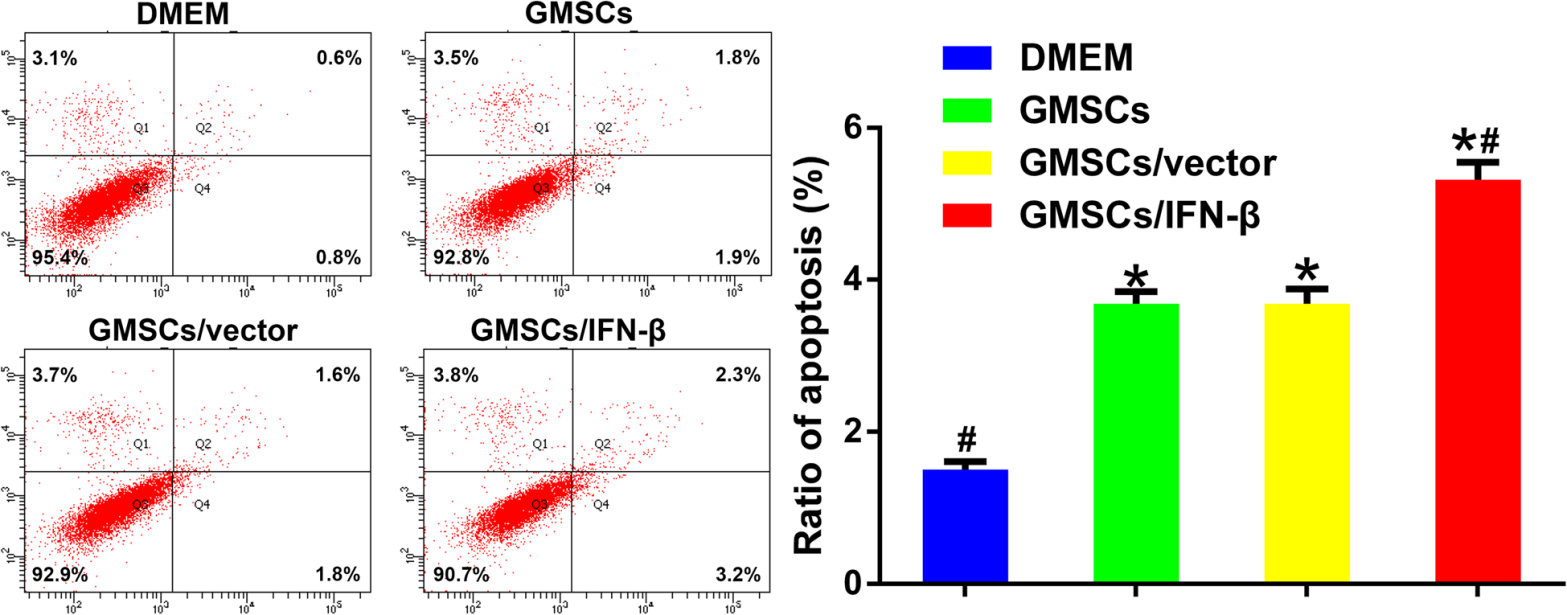
GMSCs/IFN-β promoted the apoptosis of CAL27 cells in vitro. Flow cytometry analysis showed that GMSCs/IFN-β significantly promoted cell apoptosis compared with the DMEM, GMSCs or GMSCs/vector group. All data are shown as mean ± SD. *P < 0.01 versus the DMEM group, ^#^*P* < 0.01 versus the GMSCs/vector group

To investigate the mechanism that mediates the proliferation inhibition function of GMSCs, GMSCs/vector, and GMSCs/IFN-β, flow cytometry analyses of cell apoptosis were performed. The results showed that the percentage of apoptotic cells [early apoptotic (Annexin V-FITC^+^/PI^−^) + late apoptotic cells (Annexin V-FITC^+^/PI^+^)] in GMSCs (3.68 ± 0.160%), GMSCs/vector (3.68 ± 0.194%), and GMSCs/IFN-β groups (5.32 ± 0.231%) was significantly increased compared with the control group (1.50 ± 0.110%, *P* < 0.01). GMSCs/IFN-β induced CAL27 cell apoptosis more potently than the GMSCs or GMSCs/vector group (*P* < 0.01), but no significant difference was found between the GMSCs and GMSCs/vector groups (Fig. 4).

### GMSCs/IFN-β significantly attenuated tumor growth in vivo

**Fig. 5:**
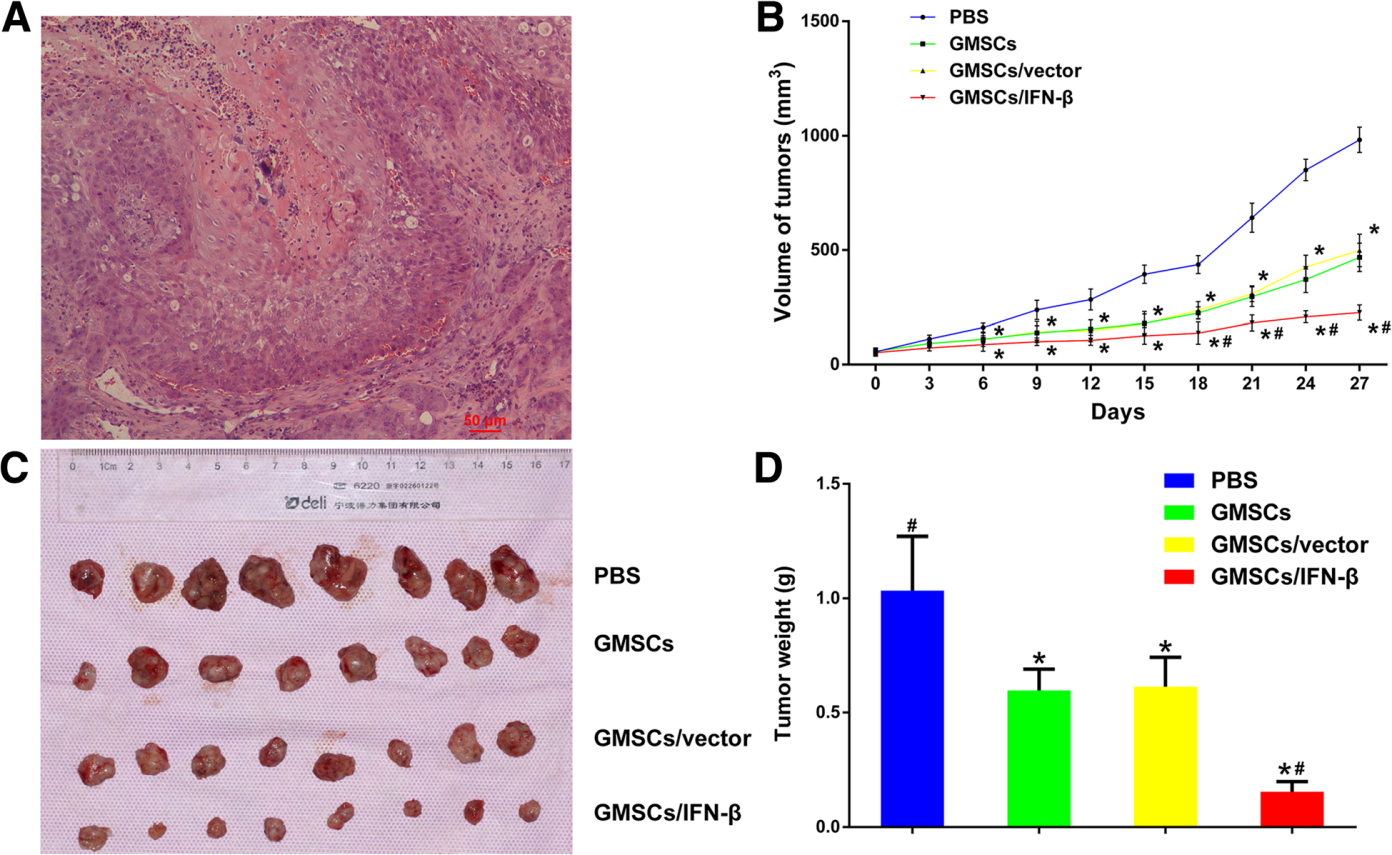
GMSCs/IFN-β attenuated tumor growth in vivo. **a** H&E staining for TSCC xenografted tumor nodules excised from experimental nude mice. **b** Representative graph of tumor growth and mean tumor volume at different time points during 4 weeks after inoculation are shown. GMSCs, GMSCs/vector, and GMSCs/IFN-β significantly decreased the tumor sizes compared with the PBS group, and especially, GMSCs/IFN-β treatment caused the most decreased tumor size in mice. **c** At the end point of treatment, mice were anesthetized and sacrificed. Then tumors were dissected and photographed. **d** Tumors of each group were collected and weighed. GMSCs/IFN-β treatment caused the most decreased tumor weight in mice compared with the PBS, GMSCs, or GMSCs/vector group. All data are shown as mean ± SD. *P < 0.05 versus the PBS group, ^#^*P* < 0.05 versus the GMSCs/vector group

In this study, the in vivo TSCC xenograft model was established as evidenced by H&E staining (Fig. 5a). Each experimental mouse bearing TSCC treated with PBS, GMSCs, GMSCs/vector, or GMSCs/IFN-β survived till 4 weeks. Tumor volumes of the GMSCs, GMSCs/vector, and GMSCs/IFN-β groups began to exhibit conspicuous difference compared with the PBS group in tumor growth from day 6, and the difference continued to expand through the experimental end point. The GMSCs/IFN-β group exhibited more potent antitumor activities compared with the PBS, GMSCs or GMSCs/vector group. The GMSCs/IFN-β group drastically reduced TSCC volume growth compared with the other three groups from day 18 (*P* < 0.05) (Fig. 5b). These results were also confirmed by the weights of the dissected tumors. The weights of the dissected tumors in the GMSCs (0.596 ± 0.0941 g), GMSCs/vector (0.614 ± 0.128 g), and GMSCs/IFN-β groups (0.155 ± 0.0444 g) were significantly decreased compared with the PBS group (1.03 ± 0.237 g) (*P* < 0.01) (Fig. 5c, d). Moreover, the GMSCs/IFN-β group induced more tumor weight decrease in mice compared with the GMSCs or GMSCs/vector group (*P* < 0.01). The volumes and weights of tumors displayed no significant difference between GMSCs and GMSCs/vector groups.

### GMSCs/IFN-β markedly attenuated cell division in tumor tissues

**Fig. 6:**
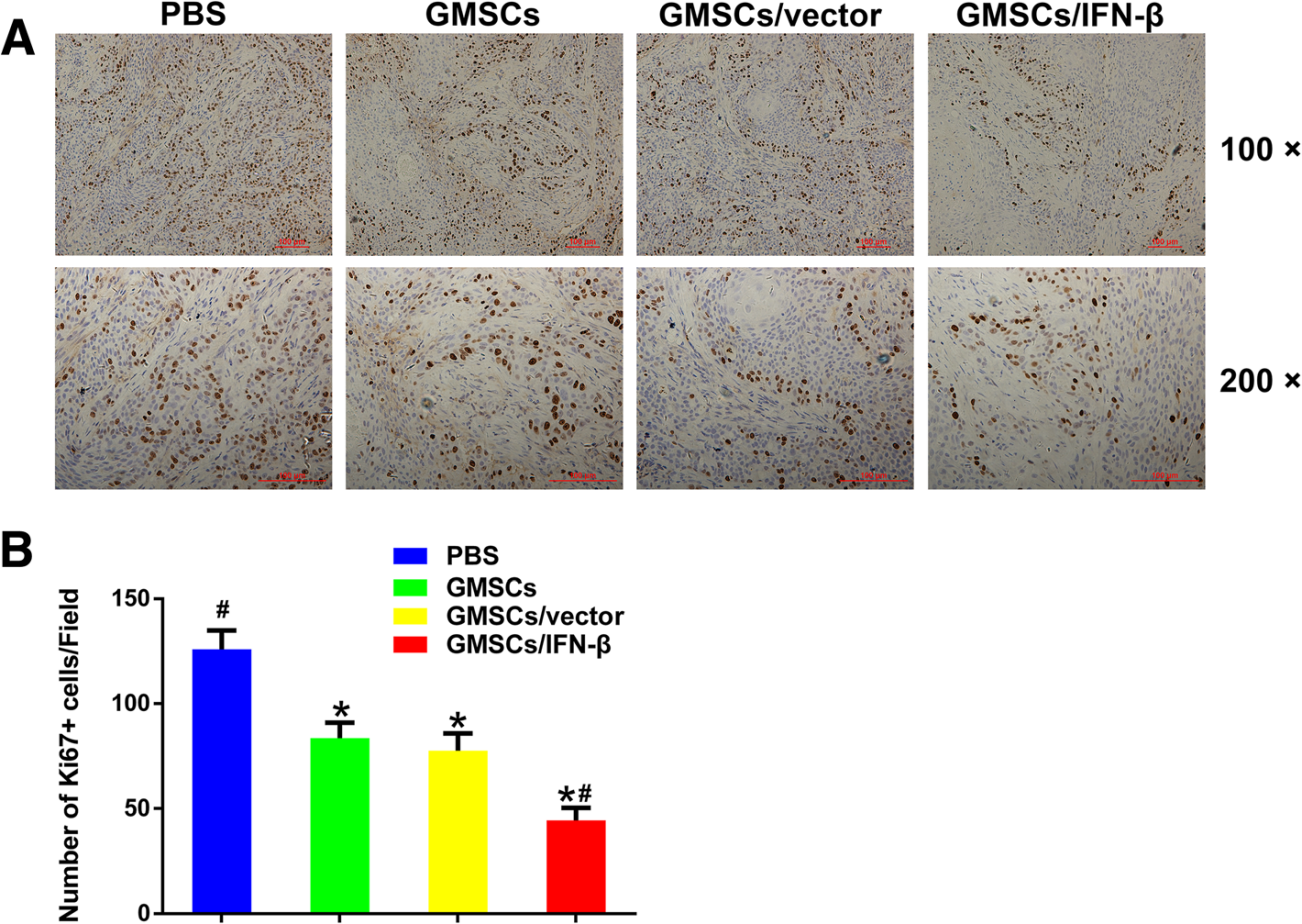
Immunohistochemical staining and quantification of the expression of Ki67 in TSCC lesion. **a** Immunohistochemical staining of Ki67 in the PBS, GMSCs, GMSCs/vector, and GMSCs/IFN-β groups of TSCC lesion. **b** Quantitative analysis of the number of Ki67-positive cells in four groups. The number of Ki67-positive cells in the GMSCs/IFN-β group was lesser than in the PBS, GMSCs, or GMSCs/vector group. All data were shown as mean ± SD. *P < 0.05 versus the PBS group, ^#^*P* < 0.05 versus the GMSCs/vector group

To evaluate the effect of different treatments on the proliferation of tumor cells, the number of Ki67-positive cells in tumor tissues was determined. The results indicated that the number of Ki67-positive cells was significantly higher in the PBS group (125.9 ± 9.04 cells/field) than in the GMSCs (83.6 ± 7.28 cells/field), GMSCs/vector (77.5 ± 8.26 cells/field), or GMSCs/IFN-β group (44.5 ± 5.84 cells/field) (*P* < 0.05). The positive expression cells of Ki67 displayed no significant difference between the GMSCs and GMSCs/vector groups. The GMSCs/IFN-β group contained the lowest Ki67-positive cells and exhibited the strongest inhibition of tumor cell proliferation compared with the PBS, GMSCs, or GMSCs/vector group (*P* < 0.05) (Fig. 6). The result suggested that GMSCs/IFN-β effectively inhibited the growth of TSCC, which may be associated with the suppression of tumor cell proliferation.

### GMSCs/IFN-β migrating to TSCC xenografts and expressing of IFN-β in vivo

**Fig. 7:**
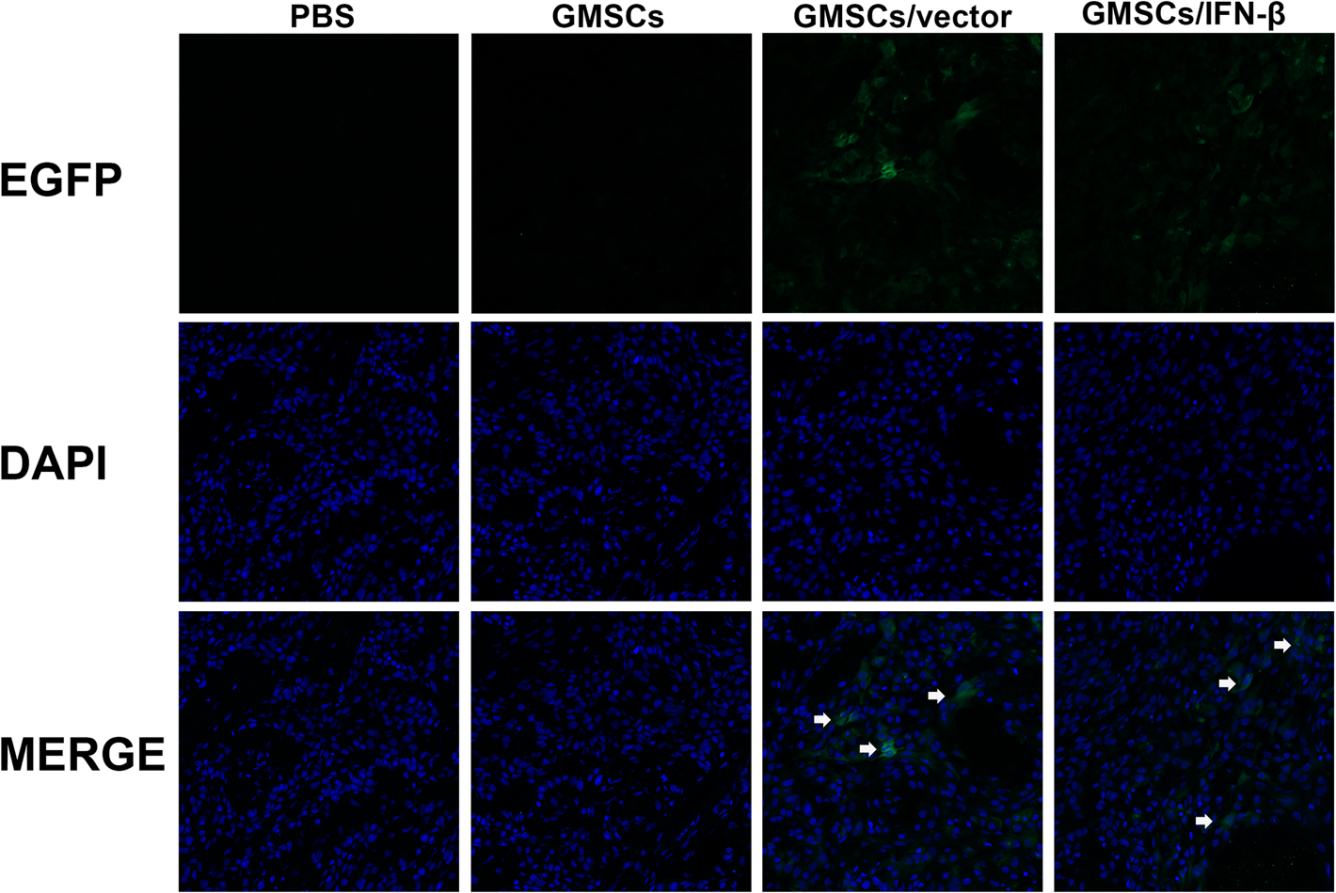
Immunofluorescence analyses of GMSCs homing to tumors in the TSCC lesion. GMSCs/vector and GMSCs/IFN-β had strong green fluorescence expression prior to in vivo administration. Green fluorescence (white arrow) was observed under confocal laser scanning microscopy in experimental nude mice which received EGFP-labeled GMSCs/vector or GMSCs/IFN-β

**Fig. 8:**
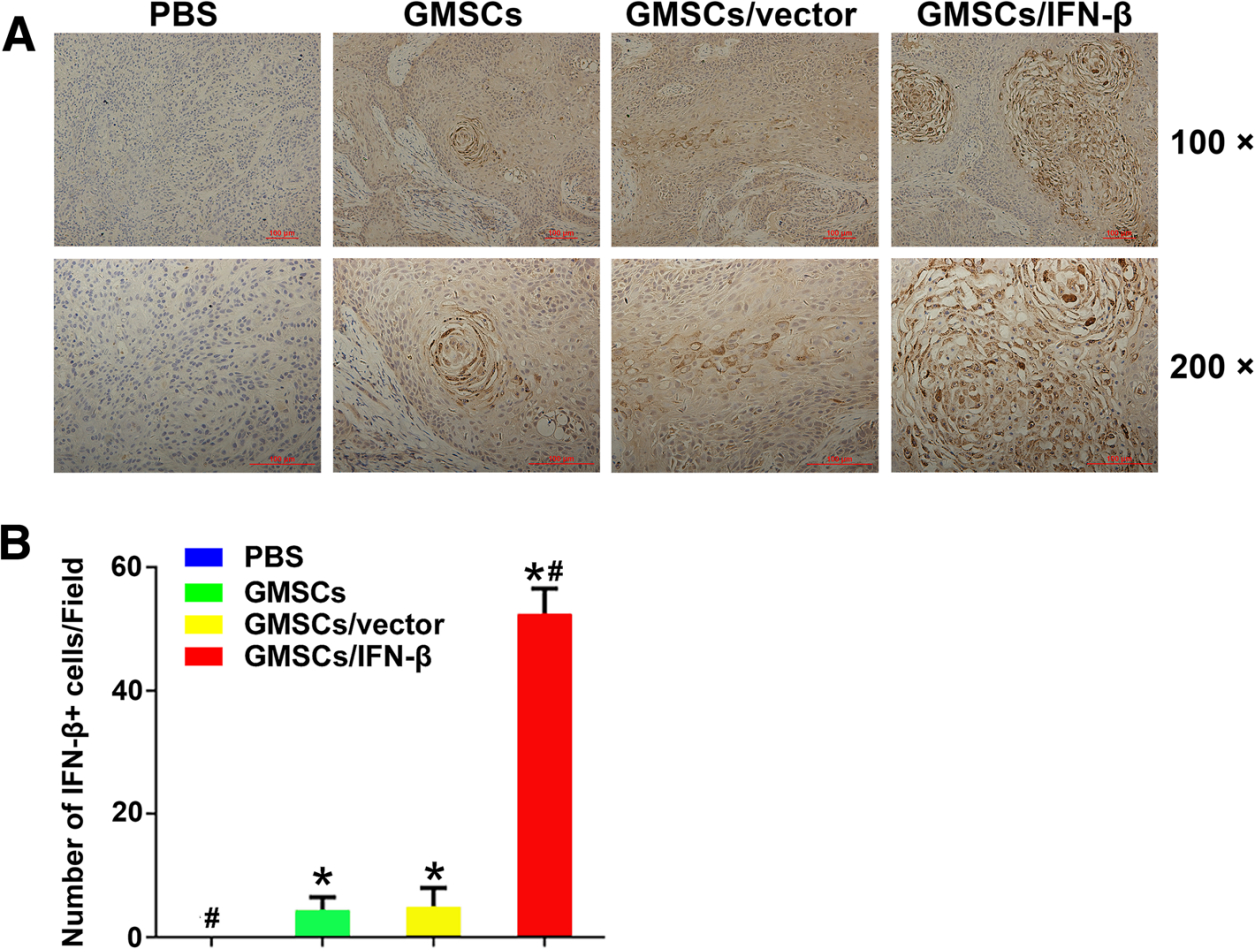
Immunohistochemical staining and quantification of the expression of IFN-β in TSCC lesion. **a** Immunohistochemical staining of IFN-β in the PBS, GMSCs, GMSCs/vector, and GMSCs/IFN-β groups of TSCC lesion. **b** Quantitative analysis of the number of IFN-β-positive cells in four groups. The number of IFN-β-positive cells in the GMSCs/IFN-β group was larger than that in the PBS, GMSCs, or GMSCs/vector group. All data were shown as mean ± SD. *P < 0.05 versus the PBS group, ^#^*P* < 0.01 versus the GMSCs/vector group

To investigate whether GMSCs/IFN-β could migrate and engraft to TSCC xenografts, GMSCs/vector and GMSCs/IFN-β were labeled with EGFP prior to in vivo administration. The tumor tissues were harvested, and the sections were made after the nude mice sacrificed. Green fluorescence was observed under confocal laser scanning microscopy in experimental nude mice which received GMSCs/vector or GMSCs/IFN-β (Fig. 7). Microscopic analysis of the tumor section provided evidence for the migration of GMSCs to the TSCC xenograft as shown in Fig. 7. Expression of IFN-β in GMSCs/IFN-β that migrated to the tumor was also confirmed by staining with an IFN-β antibody. Immunohistochemical staining results demonstrated that the tumor tissues expressed IFN-β in the GMSCs, GMSCs/vectors and GMSCs/IFN-β groups. The number of IFN-β-positive cells in the GMSCs/IFN-β group (53.2 ± 4.05 cells/field) was larger than that in the PBS (0 cells/field), GMSCs (4.20 ± 1.55 cells/field), or GMSCs/vector group (5.20 ± 2.41 cells/field) (*P* < 0.01) (Fig. 8). The expression of IFN-β in TSCC xenograft displayed no significant difference between the GMSCs and GMSCs/vector groups. The observation indicated that exogenous GMSCs can migrate and engraft to TSCC tissues in vivo.

## Discussion

IFN-β gene-modified MSCs have been demonstrated to possess consistent anticancer effects for multiple tumors, such as bronchioloalveolar carcinoma, ovarian carcinoma, breast cancer, pancreatic cancer, and prostate cancer [15, 22–28]. However, to our limited knowledge, there has been no literature reported on TSCC. In this study, we found that GMSCs/IFN-β inhibited the growth and proliferation of TSCC cell line CAL27 cells and induced cell apoptosis in vitro. Systemic administration of GMSCs/IFN-β could migrate to TSCC xenografts and dramatically attenuate tumor growth in vivo.

IFN-β is one of the classic tumor suppressors, which has been proven to significantly inhibit tumor cell growth and induce cell apoptosis in vitro [3, 4]. However, daily subcutaneous or intraperitoneal injection of large doses of IFN-β has not exhibited an effective antitumor effect in the animal xenograft models [10, 22]. The short half-life of IFN-β after routine systematic administration, thereby making it difficult to generate and maintain a therapeutic dose in tumor sites, leads to failing to inhibit tumor growth in vivo [7, 8]. Although different studies show inconsistent, even opposite effects of MSCs on tumor growth [29–31], there is a consensus that MSCs have a preferable tropism toward tumor niche which makes them ideal vector candidates for targeted antitumor therapy. IFN-β gene-modified MSCs displayed consistent anticancer effects for its long-term IFN-β expression capability [14, 25]. Here, our study likewise revealed that intravenous injection of GMSCs/IFN-β could migrate to TSCC xenografts and significantly inhibit tumor growth in terms of tumor volumes and weights. To analyze the reason why GMSCs/IFN-β inhibited tumor growth, the in vitro effects of CM from GMSCs/IFN-β on tumor cell behaviors and Ki67 expression in TSCC xenografts were determined. The results showed that CM from GMSCs/IFN-β significantly inhibited CAL27 cell proliferation and induced cell apoptosis in vitro. Ki67 is a nuclear proliferating antigen located in the nucleus and closely related to cell proliferation. It is highly expressed in various tumor tissues, and its expression level is closely related to many tumor differentiation, metastasis, and prognosis. Therefore, Ki67 can be used as a tumor cell proliferation and tumor diagnostic marker. The Ki67 expression in TSCC xenograft was also significantly decreased in the GMSCs/IFN-β group compared with that in the GMSCs or GMSCs/vector group. To further determine which component of GMSCs/IFN-β plays a role, IFN-β expressions in CM and TSCC xenografts were detected. The result revealed that IFN-β concentration in GMSCs/IFN-β group was significantly higher than that in the GMSCs or GMSCs/vector group in vitro. The in vivo expression level of IFN-β secreted by GMSCs/IFN-β was significantly higher than that of GMSCs or GMSCs/vector, which was consistent with the in vitro results. All these results suggest that GMSCs/IFN-β inhibit cell proliferation and induce cell apoptosis to suppress TSCC xenograft growth.

The use of BMSCs as a gene delivery vehicle for tumor treatment has been extensively investigated over recent decades. In addition, adipose tissue-derived mesenchymal stromal cells (AT-MSCs) and human umbilical cord-derived stem cells (hUCMSCs) have recently proven to serve as alternative sources of MSCs [15, 16, 32]. These MSCs exert a promotive or inhibitive effect on varied tumors [29–31]. The exact mechanisms of discrepancies surrounding the ability of MSCs to either promote or inhibit tumor progression have been largely unclear, but tissue source, individual secretome, nature of interactions with cancer cells and immune cells, type of cancer or cancer cell lines, and experimental conditions are considered to be the associated factors [33]. Though gene-modified MSCs displayed consistent anticancer effect, selecting those MSCs with inhibitive potential on tumor growth as vector candidates should be a better choice. Ji et al. indicated that GMSCs can suppress oral cancer cell growth in vitro and in vivo, suggested that GMSCs have a potential use in the management of oral dysplasia and oral cancer [18]. In our study, CM from GMSCs significantly inhibited the growth and proliferation of TSCC cell line CAL27 cells and induced cell apoptosis in vitro*.* Moreover, GMSCs given by intravenous injection could migrate to TSCC xenografts and exert significant inhibition on tumor growth. Given that the antitumor activity along with accessibility and availability of human gingival tissues and stronger proliferation ability than BMSCs [19, 20], GMSCs may be promising candidates for stem cell-based therapeutic genes delivery.

A potential application for therapeutic MSCs may be in the adjuvant setting for localized treatment of residual disease following surgery or radiotherapy. This could be particularly useful when extensive surgical resection or large radiation fields are difficult or associated with significant risks [34, 35]. Local direct delivery to the site of action obviously circumvents limitations associated with homing efficiency, and the high degree of overall safety observed in clinical trials using MSCs to date in other disease settings makes this strategy feasible.

## Conclusions

In summary, IFN-β gene-modified GMSCs inhibit the proliferation of TSCC cells in vitro and in vivo. These results suggest that the delivery of IFN-β by GMSCs may be an effective and promising therapeutic strategy for TSCC.

## Data Availability

The datasets used and/or analyzed during the current study are available from the corresponding author on reasonable request.

## Abbreviations

AT-MSCs: Adipose tissue-derived mesenchymal stromal cells
BM: Bone marrow
CCK8: Cell Counting Kit-8
CM: Conditioned medium
DAB: Diaminobenzidine
DAPI: 4′,6-Diamino-2-phenylindole
DMEM: Dulbecco’s modified Eagle’s medium
EGFP: Enhanced green fluorescent protein
ELISA: Enzyme-linked immunosorbent assay
FBS: Fetal bovine serum
GMSCs: Gingiva-derived mesenchymal stromal cells
GMSCs/IFN-β: IFN-β gene-modified GMSCs
H&E: Hematoxylin and eosin
hUCMSCs: Human umbilical cord-derived stem cells
IFN-β: Interferon-β
MSCs: Mesenchymal stromal cells
OSCC: Oral squamous cell carcinoma
PBS: Phosphate-buffered saline
qRT-PCR: Quantitative real-time polymerase chain reaction
TSCC: Tongue squamous cell carcinoma
